# Detection of the Fatty Acid Metabolism‐Linked Genes in Lung Adenocarcinoma as Biomarkers for Clinical Prognosis and Immunotherapeutic Targets

**DOI:** 10.1111/crj.70013

**Published:** 2024-09-25

**Authors:** Jingwei Shi, Rusong Yang, Xinyi Jiang, Kangle Zhu, Zhengcheng Liu

**Affiliations:** ^1^ Department of Thoracic Surgery, Nanjing Drum Tower Hospital Affiliated Hospital of Medical School, Nanjing University Nanjing Jiangsu Province China; ^2^ Department of Cardiovascular and Thoracic Surgery, Nanjing Drum Tower Hospital Chinese Academy of Medical Science & Peking Union Medical College, Nanjing University Nanjing Jiangsu Province China; ^3^ Department of Thoracic Surgery Nanjing Drum Tower Hospital Clinical College of Nanjing Medical University Nanjing Jiangsu Province China

**Keywords:** ACAT1, ACSL3, biomarker, fatty acid metabolism, lung adenocarcinoma, qRT‐PCR

## Abstract

**Background:**

Lung cancer, on a global scale, leads to the most common cases of cancer mortalities. Novel therapeutic approaches are urgently needed to disrupt this lethal disease. The rapid development of tumor immunology combining breakthroughs involving fatty acid metabolism brings possibilities. Directing fatty acid metabolism is supposed to help discover potential prognostic biomarkers and treatment targets for lung cancer.

**Methods:**

Through searching the GSE140797 dataset, we identified genes related to fatty acid metabolism as well as fatty acid metabolism‐related differentially expressed genes (DEGs). We applied various methods to ascertain the independent prognostic value of the DEGs. The methods we utilized entail prognostic analysis, differential expression analysis, as well as univariate and multivariate Cox regression analyses. The lasso Cox regression model was utilized in examining how DEGs correlate with the immune score, immune checkpoint, ferroptosis, methylation, and OCLR score. The expression levels of ACAT1 and ACSL3 in tissues derived from normal lung and lung adenocarcinoma (LUAD) tissues were compared by qRT‐PCR.

**Results:**

In this study, ACSL3 and ACAT1 were identified as fatty acid metabolism‐related genes utilizing independent prognostic value and as a result, the risk prognostic model was built using these factors. qRT‐PCR results implied that ACSL3 and ACAT1 expressions were upregulated and downregulated, correspondingly in tumor tissues. Additional evaluations suggested that ACSL3 and ACAT1 were affirmed to be remarkably correlated with the immune score, methylation, immune checkpoint, OCLR score, and ferroptosis.

**Conclusions:**

ACSL3 and ACAT1 were effective prognostic biomarkers and potential immunotherapeutic targets in LUAD.

## Graphical abstract

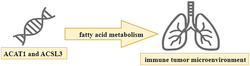

**Abbreviations**
ACAT1: Acetyl‐CoA acetyltransferase 1
ACSL3: Acyl‐CoA synthetase long chain family member 3
AUC: area under curve
CHB: chronic HBV
DEGs: differential expressed genes
FAs: fatty acids
FAS: fatty acid synthase
GO: gene ontology
GSEA: Gene Set Enrichment Analysis
HR: risk ratio
KEEG: Kyoto Encyclopedia of Genes and Genomes
LUAD: lung adenocarcinoma
OCLR: one‐class logistic regression
OS: overall survival
PCA: principal‐component analysis
RCCC: renal clear cell carcinoma
ROC: receiver operating characteristic curve
t‐SNE: t‐distributed stochastic neighbor embedding
m6A: N6‐methyladenosine
NSCLC: non‐small cell lung cancer
TME: tumor microenvironment

## 1 Introduction

Worldwide, cancer is a predominant contributor to mortalities, and cancer‐related diseases rapidly increase the burden of patients [1, 2]. The latest data indicated that lung cancer accounted for 12.4% and 18.4% of newly diagnosed cancer cases and deaths related to cancer in terms of global aspect. Non‐small cell lung cancer (NSCLC) accounts for at least 85% of entire cases of lung cancer. It is without doubt that adenocarcinoma is the most prevalent subtype in the histological classifications [3]. Lung adenocarcinoma (LUAD) is an extremely occult disease since a large number of patients have lost the opportunity of operation due to advanced stage when diagnosed [3]. Patients with lung cancer eventually present a dismal prognosis and the five‐year survival rate is very low. In addition to surgical treatment, targeted therapy is the most effective option for restraining the development of LUAD [4]. However, the efficacy rate of targeted medicines is circumscribed because of substantial individual variation [5]. Therefore, it is imperative to identify more sensitive biomarkers to detect LUAD and to explore potential targets for novel therapeutic schemes treating LUAD.

Researchers proposed that tumor is a systemic disease [6]. Tumor onset and progression are influenced by numerous metabolic pathways [7]. In addition to protein metabolism, tumor cells can alter the metabolism of carbohydrates and lipids to sustain the rapid multiplication [8]. Metabolic reprogramming stands out as a distinctive feature of malignancies as tumor cells have the talent of adapting to the surroundings and exhibit uncontrolled proliferation even when nutrients are inadequate [7, 8]. The association of tumor development with lipid metabolism has drawn the attention of researchers. It is believed that the elevation in neonatal fat is a novel characteristic of several invasive cancers including lung cancer [9, 10]. The rate of lipid metabolism varies in the emergence and development of distinct cancers. The changes in lipid levels in peripheral blood may have implications for assessing disease progression, therapeutic efficacy, and prognosis. Fatty acid metabolism, which is critical for numerous biological processes like cell membrane creation, storage of energy, and the production of signaling molecules in tumorigenesis, is garnering considerable interest [11, 12]. Qi et al. found that the unique expression pattern of genes participating in fatty acid catabolic metabolism is linked to malignancy, prognosis, and immunological phenotype in glioma [13]. Activating the nuclear factor KB signaling induces lymph node metastases among patients with cervical cancer that have enhanced lipolysis as well as fatty acid synthesis [14]. The survival of acute myeloid leukemic cells may be improved by triggering fatty acid oxidation, resulting in the remodeling and lipolysis of adipocytes of the bone marrow [15]. Furthermore, the lung possesses a robust capacity for lipid metabolism [9, 16, 17]. Active lung cancer cell growth, followed by an increase in capillaries, results in an abnormally activated lipid metabolism in the lung. The amount of fatty acid metabolism is more closely associated with lung cancer incidence and progression. Therefore, monitoring the alteration of fatty acid metabolism in lung cancer is supposed to excavate the traces of tumor development and invasion [18].

Nevertheless, the gene set linked to fatty acid metabolism in LUAD has not been comprehensively explored yet. Through this study, we hope to determine the level of fatty acid metabolism‐linked gene expression in normal versus LUAD lung tissues. Exploring the predictive value of genes linked to the metabolism of fatty acid and their interaction with the tumor immune microenvironment can establish a foundation for the identification of attractive prognostic biomarkers as well as therapeutic targets.

## 2 Material and Methods

### 2.1 Screening of Fatty Acid Metabolism‐Related DEGs and Analysis of Microarray Data

The Gene GEO database was utilized to compare the fatty acid metabolism‐linked DEGs in LUAD. The GSE140797 dataset was chosen for further analysis (https://www.ncbi.nlm.nih.gov/geo/query/acc.cgi?acc=GSE140797) [19]. We ultimately obtained the expression profile of 92 fatty acid metabolism‐linked genes utilizing previous reports about fatty acids.

To detect DEGs, the cut‐off conditions included an adjusted p‐value <0.05, and a log‐fold change|log2FC| ≥ 1 deemed statistical significance. ImageGP created Volcano maps as well as Venn maps online.

### 2.2 Functional Enrichment Analysis of Fatty Acid Metabolism‐DEGs in LUAD

Gene ontology (GO) and Kyoto Encyclopedia of Genes and Genomes (KEGG) pathway analyses were conducted by the ClusterProfiler software package. They were deployed to ascertain the functional annotation. *p*‐Value <0.05 denoted statistical significance.

### 2.3 Survival Analysis and Validation

We executed differential and prognostic analyses via the survival package so that we could ascertain the expression in conjunction with the prognostic value of fatty acid metabolism‐linked DEGs in LUAD.

Using the Cox proportional hazards model and Kaplan–Meier (K‐M) model, the risk ratio (HR) was computed. *p* < 0.05 denoted statistical significance.

### 2.4 Fatty Acid Metabolism‐DEGs Prognostic Model Construction and Verification

Following a preliminary assessment of the DEGs that portray both prognostic value and differential expression, a univariate Cox analysis was executed on the overall survival (OS) to uncover DEGs exhibiting a remarkable prognostic value (*p* < 0.05). Then, multivariate Cox regression analysis was performed to construct a prediction model based on DEGs, and the DEGs were independent prognostic factors. Signatures were developed utilizing the coefficients that matched with the independent prognostic genes. The systemic categorization of patients within the TCGA‐LUAD dataset entailed the classification of these patients into two risk groups, that is, low and high‐risk groups, and they were determined by the risk scores that were derived from the multivariate Cox regression. The principal component analysis (PCA) and the t‐distributed stochastic neighbor embedding (t‐SNE) were utilized in the exploration of the distribution of characteristics of distinct groups with the aid of R packages. The area under the curve (AUC) of the “time receiver operating characteristic curve (ROC)” determined the effectiveness of the prognostic indicators.

### 2.5 Construction of the Nomogram

Application of the R package “rms”, resulted in the generation of both the nomogram and calibration curve. Risk Scores linked to the prognostic models served as prognostic factors to assess the 1‐, 3‐, and 5‐year OS.

### 2.6 GSEA Enrichment Analysis

The clusterprofiler package performs Gene Set Enrichment Analysis (GSEA) on the DEGs. A *p*‐value <0.05 was utilized as a screening threshold.

### 2.7 Examining How the Expression of DEGs is Related to the Immune Microenvironment

The utilization of the xCell algorithm integrated with the “immunedeconv” R package facilitated the examination of the link between the expression of DEGs and both immune cells and immune scores. An examination of the impact of levels of gene expressions on eight immune checkpoint‐linked genes was executed via the utilization of the “ggplot2” R package. Lastly, the TIDE algorithm was used to evaluate two different mechanisms of tumor immune escape using DEG markers.

### 2.8 OCLR Scores Related to DEGs in LUAD

The RNA‐seq data linked to tumors was gleaned from TCGA‐LUAD, and the computation of mRNAsi was accomplished by a one‐class logistic regression (OCLR) algorithm. In the end, the dryness index was yielded.

### 2.9 Link Between Methylation and Ferroptosis With DEGs

Based on the TCGA dataset, the third‐order RNA sequencing data of genes were derived. The “ggplot2” R package subsequently evaluated the association of fatty acid metabolism‐DEGs with m6A‐related and ferroptosis‐related genes.

### 2.10 Quantitative Reverse Transcription‐Polymerase Chain Reaction (qRT‐PCR)

The approval for this research was granted by the institutional review board of the Nanjing Chest Hospital (number of ethics approval: 2017‐KL002‐03). Additionally, all participants provided signed informed consent. TRIzol (Thermo Fisher, USA) reagent extracted total RNA. By applying the HiScript III 1st Strand cDNA Synthesis Kit (Vazyme, China), qRT‐PCR was executed on the RNA derived from each sample (2 μg) on a LightCycler 480 PCR System (Roche, USA). During the PCR process, cDNA served as a template in 20 μL of reaction volume, which contained 10 μL of PCR mixture, 2 μL of cDNA template, and 0.5 μL of forward and reverse primers, along with a reasonable amount of water. The cycling conditions for the reactions of PCR commenced with a preliminary phase that encompassed DNA denaturation at 95 °C for 30 s. It was accompanied by other phases of 45 cycles at 94 °C for 15 s, 56 °C for 30 s, and lastly 72 °C for 20 s. The analyses on each sample were triplicated. Utilizing the 2‐ΔΔCT method, the GAPDH levels in every sample were employed to standardize data obtained from the threshold cycle (CT). The expression levels of mRNA were compared with controls retrieved from normal tissues. Below is an exhibition of the sequences of primer pairs particularly designed for targeted genes:

**Table:** 

Gene	Forward primersequence (5′‐3′)	Reverse primer sequence (5′‐3′)
ACSL3	GTTTTGACACAAGGGCGCAT	AGAGGCTAAGGTGGTGTGAC
ATAC1	GGAGGCTGGTGCAGGAAATA	TGCCTTTTCAATGGCTCCCT
GAPDH	GTCTCCTCTGACTTCAACAGCG	ACCACCCTGTTGCTGTAGCCAA

### 2.11 Statistical Analysis

R software (version 4.0.2), the Perl programming language (Version 5.30.2), and multivariate Cox regression analyses executed statistical analyses, data processing, and prognostic significance assessment, respectively. *p* < 0.05 or log‐rank *p* < 0.05, denoted statistical significance.

## 3 Results

### 3.1 Identification of Fatty Acid Metabolism‐DEGs in LUAD in Comparison With Normal Lung Tissues

**FIGURE 1:**
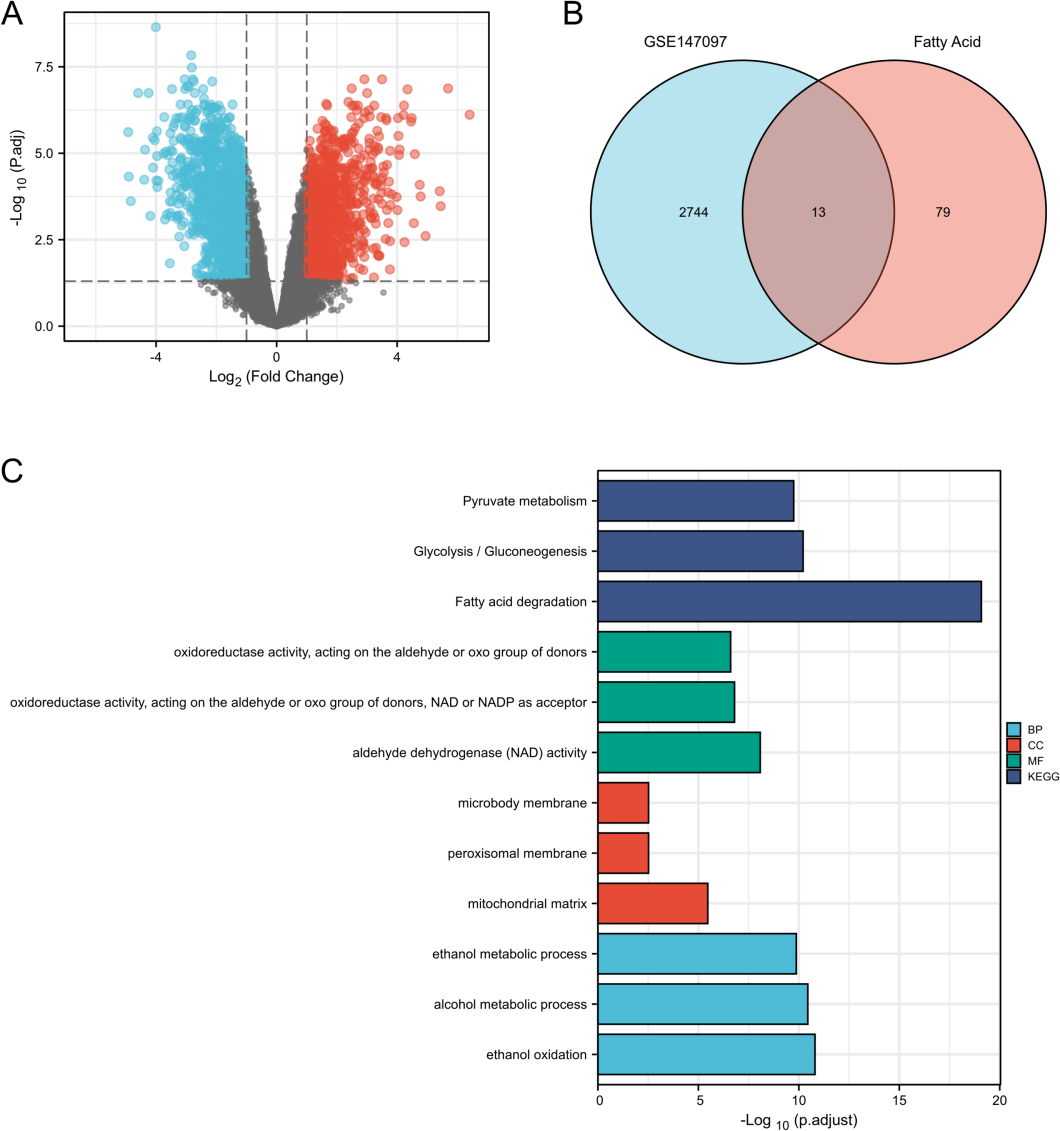
Volcano plots of DEGs between normal and cancerous lung tissues in GSE147097 samples. (A) Adjusted *p*‐value<0.05 and log2‐fold change (absolute) > 1. A total of 2757 DEGs were screened, and it was affirmed that 1233 and 1524 DEGs had upregulated and downregulated expressions, respectively. Red and blue denote upregulated and downregulated genes, respectively. The total fatty acid metabolism‐related genes derived were 92. (B) Venn diagram visualizing the 13 fatty acid metabolism‐related DEGs according to the two datasets. (C) Graph exhibiting the GO and KEEG analysis of the 13 fatty acid metabolism‐linked DEGs. The 13 fatty acid metabolism‐linked DEGs are ADH1A, ALDH3A2, ADH1B, ACADL, ALDH2, ADH5, ALDH7A1, ELOVL6, ACSL3, ACADSB, ACAT1, ACACB, and ACSS2.

In GSE140797, the volcano map showcases 1233 DEGs with an upregulated expression and 1524 DEGs with a downregulated expression, correspondingly (Figure 1A). Subsequently, 92 fatty acid metabolism‐linked genes were examined via the Venn diagram, and 13 co‐expressed genes were distinguished according to the two datasets: ADH1A, ALDH3A2, ADH1B, ACADL, ALDH2, ADH5, ALDH7A1, ELOVL6, ACSL3, ACADSB, ACAT1, ACACB, and ACSS2 (Figure 1B). In GO and KEGG analyses, for the 13 co‐expressed genes, it was affirmed that their functions are predominantly centered on “fatty acid degradation” (Figure 1C).

### 3.2 Differential Expression and Survival Analyses of Fatty Acid Metabolism‐DEGs in LUAD

**FIGURE 2:**
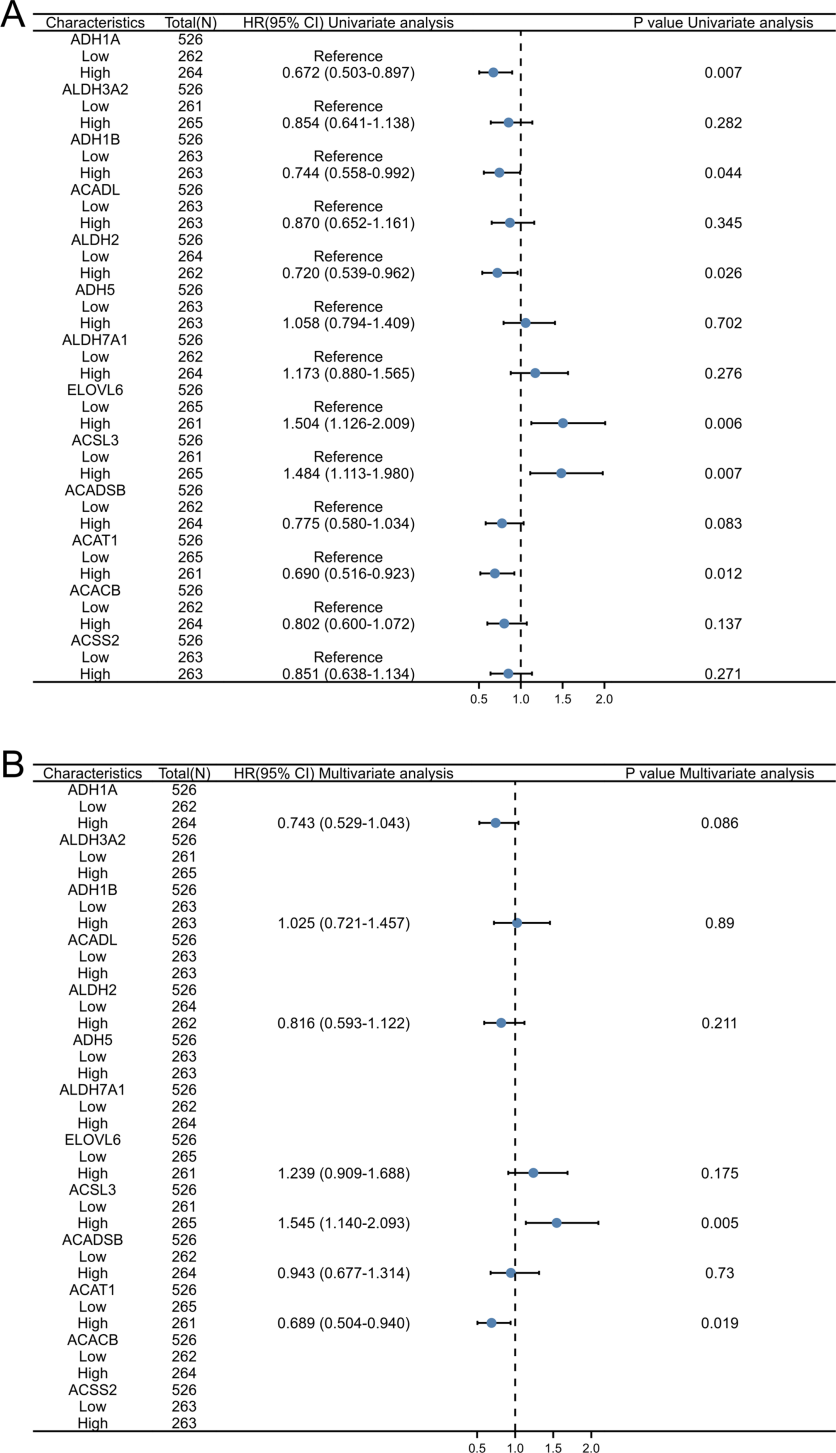
The forest plots for both univariate and multivariate Cox regression analysis (A) Forest plot manifesting the findings of the univariate Cox regression analyses of the 13 fatty acid metabolism‐related DEGs in TCGA‐LUAD. (B) The forest plot exhibiting the findings of the multivariate Cox regression analyses of the 13 fatty acid metabolism‐linked DEGs in TCGA‐LUAD. ACAT1 and ACSL3 are significantly altered in both of the analyses.

**FIGURE 3:**
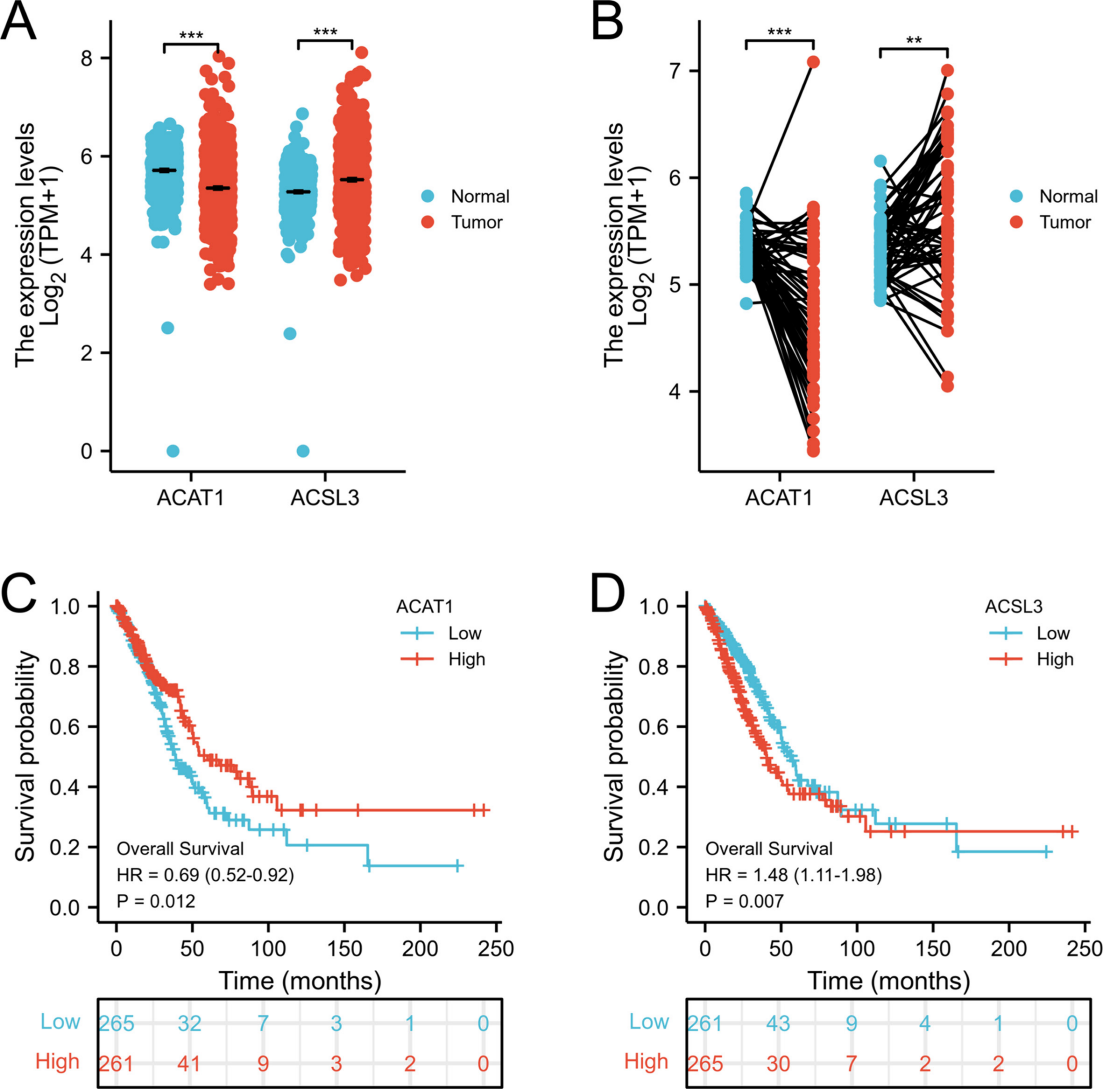
(A–B) Comparative analysis of LUAD samples and normal tissues executed to ascertain the expression profile of the two fatty acid metabolism‐linked DEGs. (C–D) Kaplan–Meier plots showing ACAT1 and ACSL3 with prognostic value. *p < 0.05, **p < 0.01, and ***p < 0.001.

The correlation between prognosis and the 13 DEGs was explored utilizing univariate Cox regression analysis and multivariate Cox regression analysis as illustrated in Figure 2A and Figure 2B. Results show that ACAT1 and ACSL3 are independent prognostic factors for LUAD. Through the screening of the TCGA‐LUAD database, expression levels of ACAT1 and ACSL3 were compared in normal lung tissues with LUAD tissues. In comparison to ACAT1, it was affirmed that ACSL3 in tumor tissues was up‐regulated (Figure 3A–B). Furthermore, K‐M model analysis displayed that the high expression of ACAT1 is linked to a favorable prognosis (Figure 3C), whereas the high expression of ACSL3 represents a poor prognosis (Figure 3D).

### 3.3 Fatty Acid Metabolism‐Related DEGs Prognostic Risk Model Construction and Validation

**FIGURE 4:**
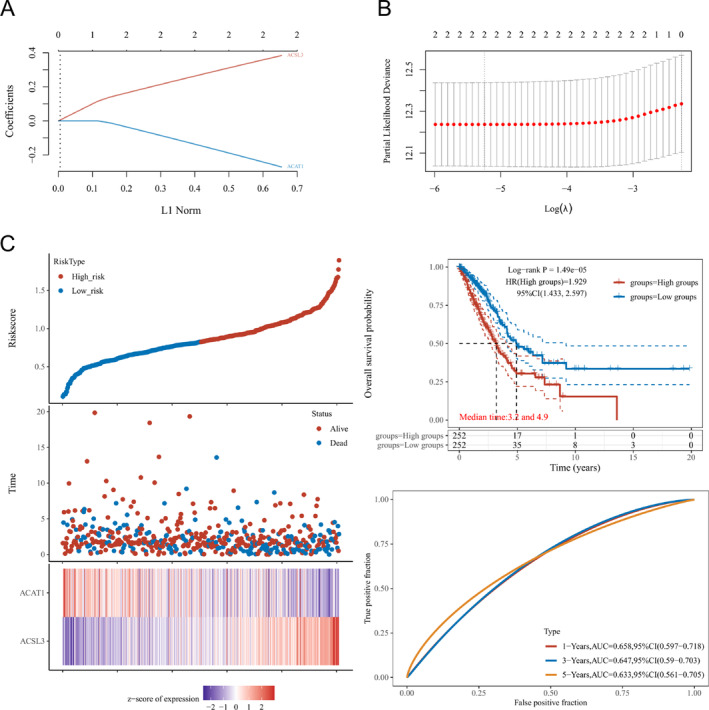
(A–B) The computations of the model as per the multivariate Cox regression analyses. (C) The prognostic model was evaluated utilizing target gene expression heat map, survival time and status, and 1‐, 3‐, and 5‐year OS. lambda.min = 0.0053. Riskscore = (0.3737) * ACSL3 + (−0.2587) * ACAT1. A nomogram was used to anticipate 1‐, 3‐, and 5‐year OS in the whole TCGA cohort. As manifested, C‐index works as 0.623. We additionally discovered that the 1‐, 3‐, and 5‐year OS of the nomogram concurred with the projected probability of the calibration curve as manifested.

Lasso Cox regression constructed a prognostic model of risks related to DEGs, with lambda.min = 0.0053 and Riskscore = (0.3737) * ACSL3 + (−0.2587) * ACAT1 (Figure 4A–B) in this study. High‐ and low‐risk groups of patients were assigned utilizing the median risk score (50%). The prognostic model, denoted by an HR = 1.929, could function as a risk factor model. Based on Figure 4C, it was affirmed that the high‐risk group's median survival time was considerably less than that of the low‐risk group. ROC was utilized to ascertain the efficiency of prognostic prediction of the model, and the findings affirmed that the AUC values were 0.658, 0.647, and 0.633 for 1‐, 3‐, and 5‐year OS, correspondingly as visualized in Figure 4C.

### 3.4 Link Between the Expression of Immune Infiltrating Cells in LUAD Tissues and DEGs Linked to Fatty Acid Metabolism

**FIGURE 5:**
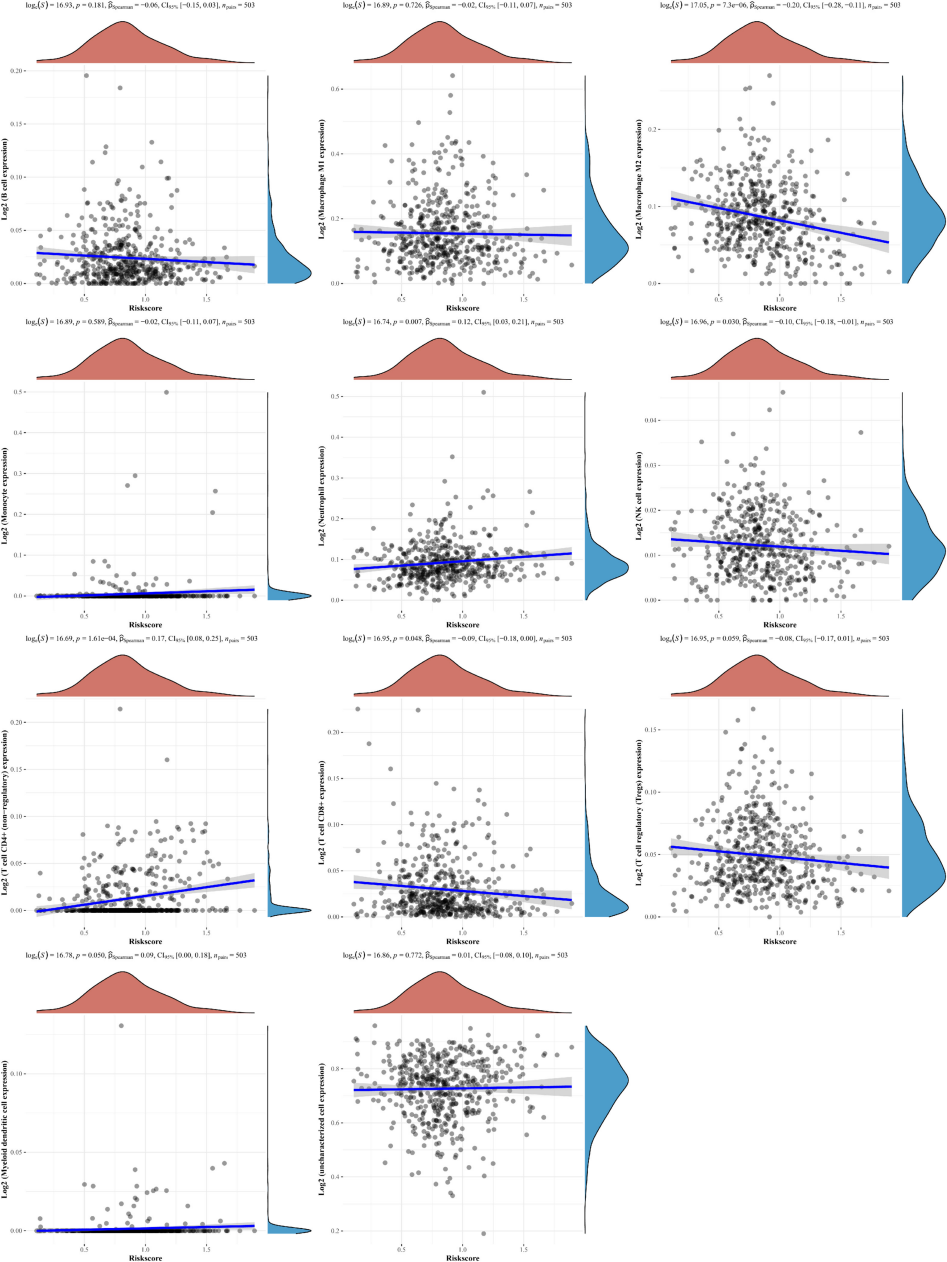
Spearman correlation analysis assessing the association between risk score and immune score.

Spearman correlation between immune score and prognosis model score was analyzed and is shown in Figure 5 It is presented that the prognosis model is remarkably linked to the expression levels of diverse immune infiltrating cells, such as Macrophage M2 cell, Neutrophil cell, NK cell, CD4 + T cell, and CD8 + T cell.

The population of LUAD in the TCGA‐LUAD database was classified into fatty acid metabolism‐related DEGs low expression group (G1) and fatty acid metabolism‐related DEGs high expression group (G2), and we determined the link between the expression of immune‐infiltrating cells and fatty acid metabolism‐related DEGs. It is determined that ACAT1 and ACSL3 are substantially linked to the expression levels of the immune infiltrating cells. The expression levels of lymphoid progenitor, B cell memory, B cells, and T cell NK are remarkably correlated with ACAT1 and ACSL3. Findings imply that the aforementioned cells might be linked to the progression of LUAD.

### 3.5 Link Between the Expression of Immune Checkpoint in LUAD Tissues and Fatty Acid Metabolism‐Linked DEGs

**FIGURE 6:**
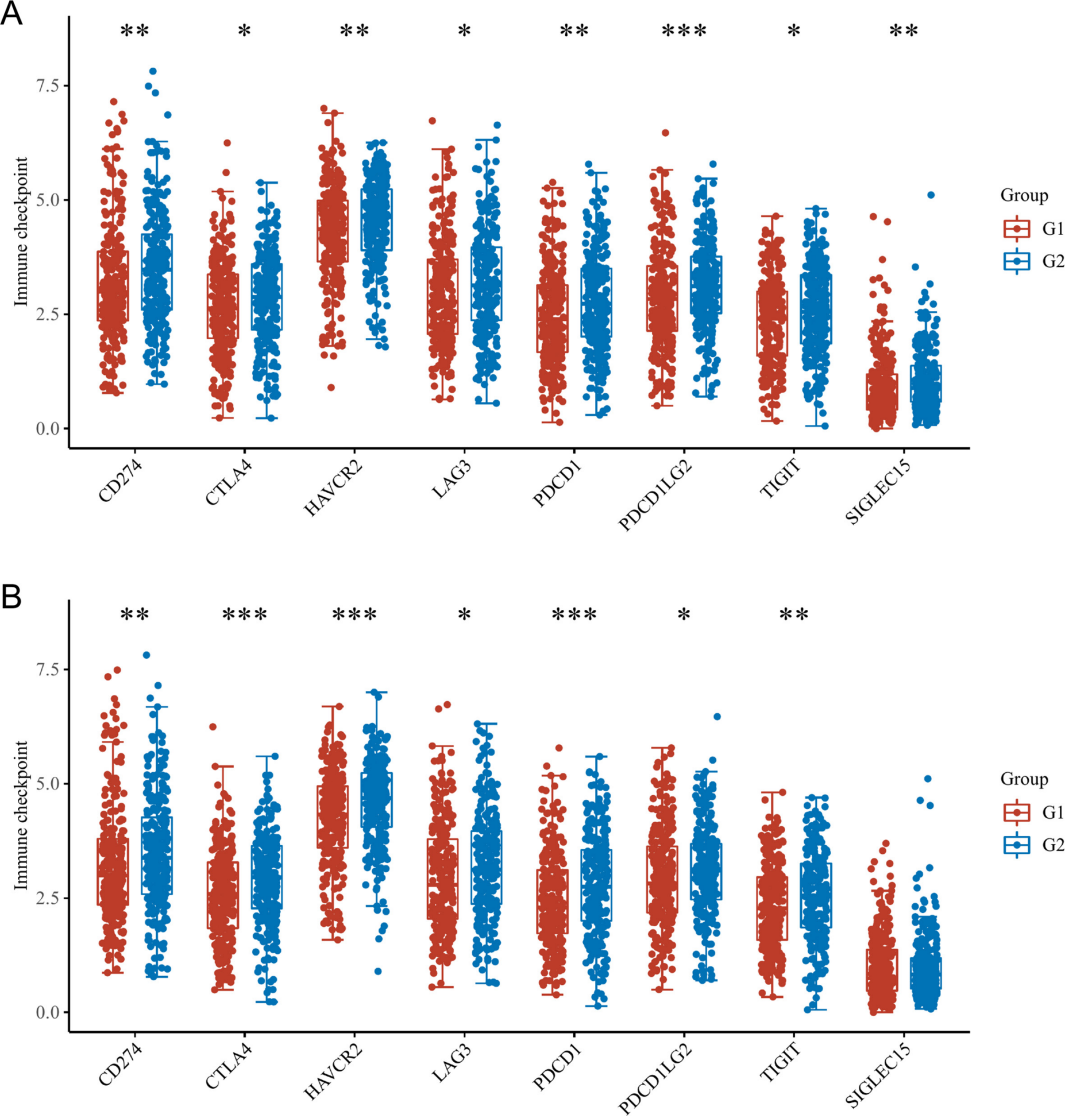
The variation in expression of immune checkpoint in LUAD tissues with high and low ACAT1 (A) and ACSL3 (B) gene expression. G1 and G2 denote high‐ and low‐expression groups, correspondingly. *p < 0.05, **p < 0.01, and ***p < 0.001.

Our study aimed at uncovering targeted therapies for individuals with LUAD, and as part of the analysis, we examined statistically the link between the expression levels of immune checkpoints in LUAD tissues and the fatty acid metabolism‐linked DEGs. The findings implied that CD274, CTLA4, LAG3, HAVCR2, PDCD1, PDCD1LG2, SIGLEC15, and TIGIT are substantially correlated with ACAT1 (Figure 6A), while CTLA4, PDCD1LG2 HAVCR2, LAG3, CD274, PDCD1, and TIGIT are remarkably linked to ACSL3 (Figure 6B). HAVCR2, CTLA4, CD274, PDCD1, LAG3, PDCD1LG2, and TIGIT have appeared twice as evidenced above. The conjecture is that these checkpoints could serve as valuable indicators in both the treatment and diagnosis of LUAD because of their sensitivity.

### 3.6 Examination of the OCLR Scores of Fatty Acid Metabolism‐Related DEGs in LUAD

**FIGURE 7:**
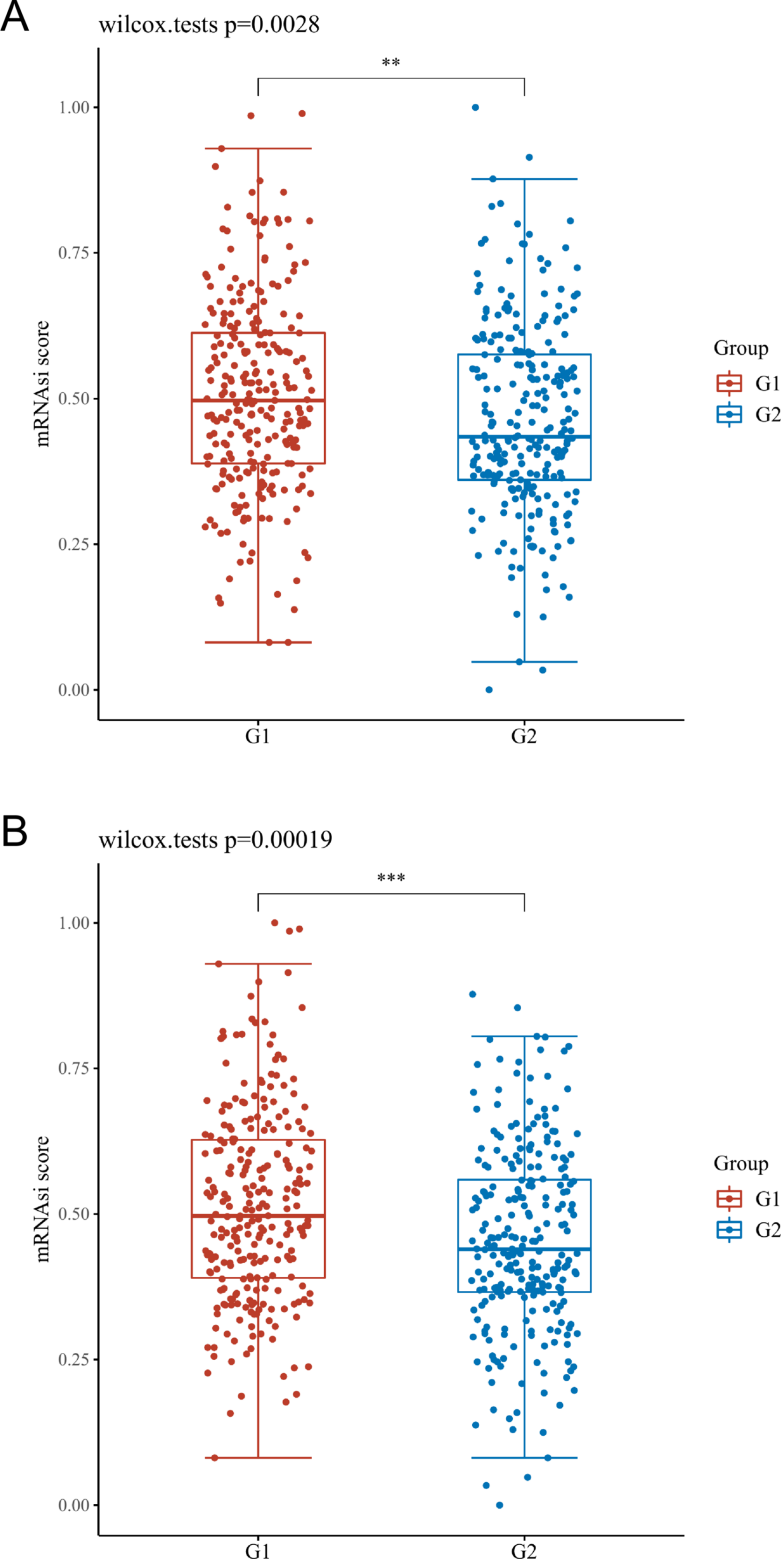
Examination of the OCLR scores of fatty acid metabolism‐linked DEGs in LUAD. Scatter diagram exhibiting the link between ACAT1 (A) and ACSL3 (B) and OCLR score in LUAD. The horizontal axis, vertical axis, G1, and G2 denote gene expression distribution, the OCLR score distribution, the high expression group, and the low expression group, correspondingly. *p < 0.05, **p < 0.01, and *** *p* < 0.001.

By assessing the OCLR scores, expression levels of ACAT1 and ACSL3 are found to be remarkably distinct from the dryness extent of LUAD (Figure 7A–B). The findings suggest that ACAT1 and ACSL3 might have a function in the spectrum of similarity between stem cells and LUAD cells and thus impact the biological process and the dedifferentiation of tumors.

### 3.7 Link Between the Expression of Fatty Acid Metabolism‐Related DEGs With Methylation and Ferroptosis

Additionally, we examined the link between m6A methylation‐linked genes and fatty acid metabolism‐linked DEGs. Results indicate that ACAT1 and ACSL3 are remarkably linked to many methylated genes.

The findings reveal that the expression of fatty acid metabolism‐linked DEGs is associated with the expression levels of several genes linked to ferroptosis when we deployed the same analysis method.

### 3.8 qRT‐PCR Gene Expression Verification

**FIGURE 8:**
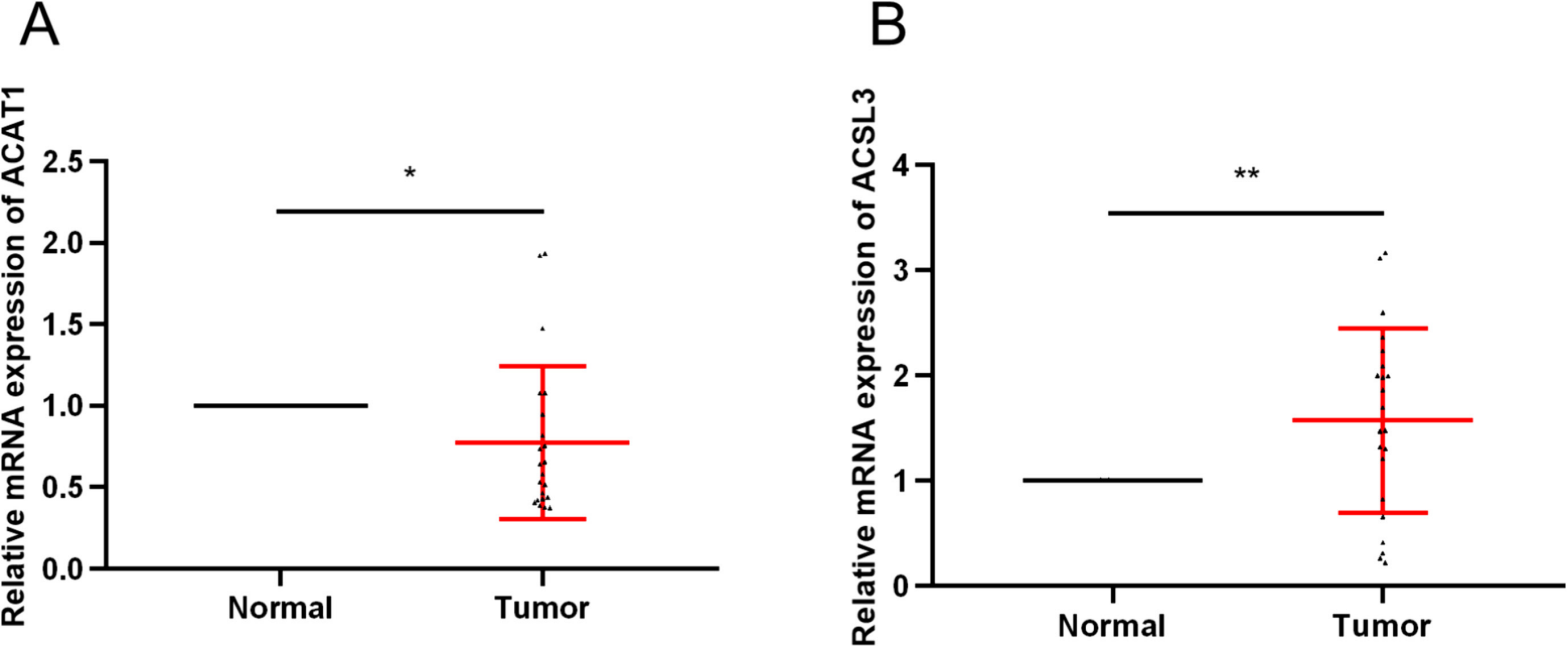
ACAT1 and ACSL3 qRT‐PCR gene expression.

The expression levels of ACAT1 and ACSL3 in normal lung tissues and LUAD tissues were compared by qRT‐PCR. The findings affirmed that ACSL3 and ACAT1 expressions were upregulated and downregulated, correspondingly in tumor tissues (Figure 8A–B).

## 4 Discussion

Although histology‐based chemoradiotherapies continue to play a fundamental role in treating advanced lung cancer, immunotherapies and targeted therapies based on specific biomarkers are gaining increasing importance in comprehensive programs [3, 4, 5]. Immune‐targeted therapies like checkpoint inhibitors are rapidly developed [3, 4]. They are capable of significantly improving the OS time of lung cancer patients. It is urgently required to increase lung cancer survival through the discovery of novel prognostic biomarkers and treatment targets.

To restrain tumor derivation and progression, it is vital to comprehend how the surrounding substance supports abnormal cell growth, and the instrumental genes involved. Douglas described the next generation of hallmarks of human tumors [8]. Independent of the core capabilities, altered metabolism is an emerging hallmark taking place in cancer [8]. Recent literature demonstrated the metabolic alterations of lipids in lung cancer onset and progression [16, 17, 18, 19, 20]. It was determined that energy metabolism including lipid metabolism reprograms to support aberrant cell growth and division in lung [16], while lipids are predominately synthesized from fatty acids (FAs). The involvement of enzymes is essential for FA uptake, synthesis, and degradation [21]. For instance, activated enzymes like ACLY, ACC, FASN, and SCD1 were highly expressed in lung cancer tissues [17], which are correlated with a worse prognosis among patients having NSCLC. Visca et al. examined the expression of fatty acid synthase (FAS) in 106 patients diagnosed with NSCLC and discovered a trend for improved survival in stage I patients with FAS‐negative expression (*p* = 0.10) [20]. An identical finding was shown by Wang et al. [17] where the expression of FAS was detectable in 175 specimens of patients with NSCLC. The survival rate of FAS‐positive cases was substantially lower than that of FAS‐negative cases (*p* = 0.005) [17], indicating that FAS is a positive factor for the poor prognosis of stage I patients. In this investigation, 13 fatty acid metabolism‐related genes are identified to be differentially expressed in LUAD compared to the normal tissues. These genes are primarily involved in fatty acid degradation. Investigating the correlation of these differentially expressed genes (DEGs) with lung cancer progression may pave the way to the excavation of novel biomarkers and potential treatments for LUAD.

Through Cox regression analysis and Kaplan–Meier model analysis of fatty acid metabolism‐DEGs, Acetyl‐CoA acetyltransferase 1 (ACAT1) and Acyl‐CoA synthetase long‐chain family member 3 (ACSL3) are supposed to possess the forecasting capability of predicting the prognosis of patients with LUAD. As a mitochondrial enzyme, ACAT1 plays a critical part in fatty acid metabolism by catalyzing the formation of acetoacetyl‐CoA [21]. Chen et al. determined that ACAT1 was a negative prognostic biomarker of renal clear cell carcinoma (RCCC) by data analysis and validation using qPCR analysis and immunohistochemistry staining of specimens [22]. An increase in ACAT1 was examined to suppress RCCC cell growth, colony formation, and mobility [22], whereas ACAT1 downregulation by miR‐21‐5p facilitated EMT in RCCC [23]. Herein, lower expression of ACAT1 is found in LUAD tissues while a higher level of ACAT1 indicates a better prognosis in patients. There is a possibility that ACAT1 might act as a negative predictor for LUAD. Meanwhile, ACSL3 also exhibits attractive potential as an effective positive indicator for LUAD. It was reported that ACSL3 is an essential enzyme engaged in fatty acid metabolism, esterifying FAs with CoA and participating in the cellular uptake of FAs [21, 24, 25]. Studies demonstrated that overexpression of ACSL3 was detected in various malignancies such as hepatoma, prostate cancer, and NSCLC [25, 26, 27], revealing ACSL3 as a promising protective factor of prognosis in patients with cancer. Weedon‐Fekjaer et al. reported that ACSL3, which is regulated by the activation of the liver X receptor, directly enhanced acyl‐CoA synthetase activity in human trophoblast cells [27]. As is well known, pro‐oncogene KRAS mutation frequently occurs in lung carcinoma. Mutant KRAS increased about by three‐fold the activity of an ACSL3 promoter reporter (pGL3‐ACSL3luc) depending on the mTOR signaling pathway, resulting in an upregulation of fatty acid metabolism in NSCLC [26]. Besides, the prediction efficiency of the model composed of ACAT1 and ACSL3 was well validated in this study.

One of the crucial actions of the human immune system is to monitor tissue homeostasis and eliminate damaged cells [28]. For the eradication of cell mutations and tumorigenesis, the presence of a full‐fledged immune response is essential. As an important component of the immune microenvironment, infiltrated immune as well as inflammatory cells have been demonstrated to have paradoxical roles in the development of tumors [28]. Energy metabolism not only reprograms the power consumption of aberrant cell proliferation but also fuels immune cell function and multiple immune responses. The association of fatty acid metabolism with immune conduct in cancer attracts the wide attention of researchers [29]. For instance, the inhibition of ACAT in cultured mononuclear cells derived from patients with chronic HBV (CHB) elevated CD8 + T cell proportion, resulting in the enhancement of IFNγ response [10]. Modifying the activity of ACAT1 might be a feasible method to modulate the anti‐tumor efficiency of killer T cells [29]. Additionally, Toshio Kanno et al. pointed out that members of the ACSL family were detectable in T cells and Acsbg1‐mediated acyl‐CoA synthesis played a vital role in lung Treg cell homeostasis [30]. We further verified that the altered expression of immune infiltrating cells is highly linked to fatty acid metabolism‐related DEGs, ACAT1, and ACSL3. Surprisingly, the existence of checkpoints PDCD1LG2, HAVCR2, CTLA4, LAG3, PDCD1, CD274, and TIGIT is consistent with the alteration of not only ACAT1 but also ACSL3. As an intrinsic part of the immune system, immune checkpoints offer promised targets for cancer immunotherapies. The synergistic effect of PD‐1 blockade together with ACAT inhibition was seen to be additive in 11 out of 26 patients with CHB [10]. Despite the fact that additional steps are required to analyze the strategies that rely on the combination of these checkpoint molecules with ACAT1 and ACSL3, this study demonstrates the enormous potential of targeted therapeutics for LUAD.

N6‐methyladenosine (m6A) appears to be the most frequent internal modification among kinds of epigenetic modifications in eukaryotic cells [31]. M6A modulates cell differentiation, self‐renewal, invasion, and apoptosis through modulating gene expression, thereby m6A alterations participate in the pathogenesis and progression of various cancers [32]. M6A‐related genes such as BRD4, MYC, SOCS2, and EGFR are frequently involved in the progression of cancers including LUAD [32]. Findings pointed out that m6A modifications possess the behaviors of infiltrated immune cells in the tumor microenvironment (TME), indicating its functions in diverse tumor immunity processes [33]. Herein, we demonstrated that the expression of ACAT1 and ACSL3 is remarkably linked to the levels of multiple methylated genes. Additionally, the correlation of ACAT1 and ACSL3 with ferroptosis‐related genes was also investigated in this study. Ferroptosis is essential for tissue homeostasis and the prevention of atypical hyperplasia [34]. Aberrant cell growth is inhibited by ferroptosis induced in the tumor resulting in the suppression of tumorigenesis [35]. Therapeutic and prognostic benefits of ferroptosis modulation and ferroptosis‐related genes have been determined in lung cancer [35]. Zou et al. ascertained the inhibitive effect of ferroptosis induced in NSCLC on tumor progression in vitro and in vivo [36]. Regulation of the classic ferroptosis‐repressed GSH‐dependent GPX4 signaling pathway was the main mechanism underlying [36]. Bioinformatics analysis and experimental results suggest the possible function of ferroptosis‐linked genes involved in NSCLC progression and prognosis [37, 38]. Regarding the momentous functions of fatty acid metabolism‐related DEGs in oncogenesis, it is not surprising that the expression of ACAT1 and ACSL3 is determined to be correlated with the levels of the genes related to ferroptosis.

While the study on fatty acid metabolism and its relation to lung cancer progression presents promising insights, several limitations must be considered. First, the research largely relies on correlation data, which does not establish causation. This could lead to overinterpretation of the role of genes like ACAT1 and ACSL3 without understanding the precise mechanisms by which they influence tumor behavior. Additionally, the findings are based on data from a limited number of studies and patient samples, which may not fully represent the diverse genetic and environmental factors influencing lung cancer. The reliance on histological and metabolic data might also overlook the complexities of other molecular interactions and tumor microenvironment factors. Furthermore, while the potential therapeutic implications of targeting ACAT1 and ACSL3 are highlighted, the study does not address potential side effects or the feasibility of translating these findings into clinical practice. Finally, the integration of epigenetic modifications such as m6A and their interactions with fatty acid metabolism adds another layer of complexity that is not fully explored in this research. Overall, while the study provides a valuable foundation, further research is needed to validate these findings and to explore their broader applicability and clinical relevance.

## 5 Conclusion

Overall, ACAT1 and ACSL3 are identified as fatty acid metabolism‐DEGs with validated prognostic value for patients with LUAD. The alterations of ACAT1 and ACSL3 are closely linked to the immune tumor microenvironment and progression of LUAD. We believe that targeting ACAT1 and ACSL3 is a potential choice for the development of immunotherapies treating LUAD.

## Author Contributions

J.W.S. and Z.C.L. wrote the main manuscript, J.W.S., K.L.Z. and X.Y.J. prepared Figures 1–12, and J.W.S. and Z.C.L. edited the manuscript. Each author reviewed the manuscript.

## Patient Consent for Publication

Not applicable.

## Conflicts of Interest

The authors declare no conflicts of interest.

## Data Availability

The data that support the findings of this study are openly available in geo at https://www.ncbi.nlm.nih.gov/.
